# Pain in acute hepatic porphyrias: Updates on pathophysiology and management

**DOI:** 10.3389/fneur.2022.1004125

**Published:** 2022-11-21

**Authors:** Mohamed Kazamel, Elena Pischik, Robert J. Desnick

**Affiliations:** ^1^Department of Neurology, University of Alabama at Birmingham, Birmingham, AL, United States; ^2^Department of Neurology, Consultative and Diagnostic Center With Polyclinics, St. Petersburg, Russia; ^3^Department of Genetics and Genomic Sciences, Icahn School of Medicine at Mount Sinai, New York, NY, United States

**Keywords:** δ-aminolaevulinic acid (ALA), dysautonomia, hemin, neuropathy, porphyria, siRNA

## Abstract

Acute hepatic porphyrias (AHPs) typically present with recurrent acute attacks of severe abdominal pain and acute autonomic dysfunction. While chronic symptoms were historically overlooked in the literature, recent studies have reported increased prevalence of chronic, mainly neuropathic, pain between the attacks. Here we characterize acute and chronic pain as prominent manifestations of the AHPs and discuss their pathophysiology and updated management. In addition to the severe abdominal pain, patients could experience low back pain, limb pain, and headache during acute attacks. Chronic pain between the attacks is typically neuropathic and reported mainly by patients who undergo recurrent attacks. While the acute abdominal pain during attacks is likely mediated by autonomic neuropathy, chronic pain likely represents delayed recovery of the acute neuropathy with ongoing small fiber neuropathy in addition to peripheral and/or central sensitization. δ-aminolaevulinic acid (ALA) plays a major role in acute and chronic pain *via* its neurotoxic effect, especially where the blood-nerve barrier is less restrictive or absent i.e., the autonomic ganglia, nerve roots, and free nerve endings. For earlier diagnosis, we recommend testing a spot urine porphobilinogen (PBG) analysis in any patient with recurrent severe acute abdominal pain with no obvious explanation, especially if associated with neuropathic pain, hyponatremia, autonomic dysfunction, or encephalopathy. Of note, it is mandatory to exclude AHPs in any acute painful neuropathy. Between the attacks, diagnostic testing for AHPs should be considered for patients with a past medical history of acute/subacute neuropathy, frequent emergency room visits with abdominal pain, and behavioral changes. Pain during the attacks should be treated with opiates combined with hemin infusions. Symptomatic treatment of chronic pain should start with gabapentinoids and certain antidepressants before opiates. Givosiran reduces levels of ALA and PBG and likely has long-term benefits for chronic pain, especially if started early during the course of the disease.

## Introduction

The Acute hepatic porphyrias (AHPs) are a group of inherited metabolic disorders due to the hepatic overproduction of the neurotoxic porphyrin precursors, δ-aminolaevulinic acid (ALA) and porphobilinogen (PBG) (1, 2). All AHPs have similar manifestations with recurrent acute attacks of severe abdominal pain and acute autonomic dysfunction (3, 4). Acute intermittent porphyria (AIP) is the most common AHP in Europe and the US. Variegate porphyria (VP) and hereditary coproporphyria (HCP) are rarer and may present with photosensitive skin lesions independent of the neurological manifestations (4).

Acute pain is the major manifestation of attacks, and most patients report severe abdominal pain (5, 6). Other types of pain, like back and limb pain were also reported during attacks (7, 8). The acute attacks are typically provoked by porphyrinogenic drugs that induce certain hepatic cytochrome P450 enzymes, hormonal changes in women during the luteal phase of menstrual cycle, and fasting. Over 90–95% of AIP patients who experience acute attacks are females with onset typically after puberty (9). While the prevalence of AIP pathogenic mutations in Caucasians is ~ 1/1,700 (10), the acute attacks occur in only ~ 1% (11).

The presence of chronic neurological symptoms was neglected for many years by porphyrinologists, and AHPs patients were considered “healthy” between attacks (1). However, clinical studies in the last decade reported chronic, mainly neuropathic, pain among 18% of US patients (9) and in most patients with recurrent attacks (12). The objectives of this review are to characterize pain as a prominent manifestation of the AHPs and to discuss its pathophysiology and updated management.

## Clinical presentation of pain

### During acute attacks

#### Abdominal pain

Most patients (74–100%) report severe abdominal pain (5, 6, 12). It usually lasts several hours to days and does not reach nadir before 2 days (6, 7, 13). Pain is severe enough to give an impression of an “acute catastrophe” in the abdomen. The pain character is either colicky or gnawing/aching and is commonly accompanied by nausea and vomiting (42–88%) (5). It was reported in different abdominal regions e.g., epigastrium, right iliac fossa, lower abdomen, or generalized over the whole abdomen (12). The abdominal examination and routine laboratory/radiologic workups are often unremarkable (2).

**Figure 1:**
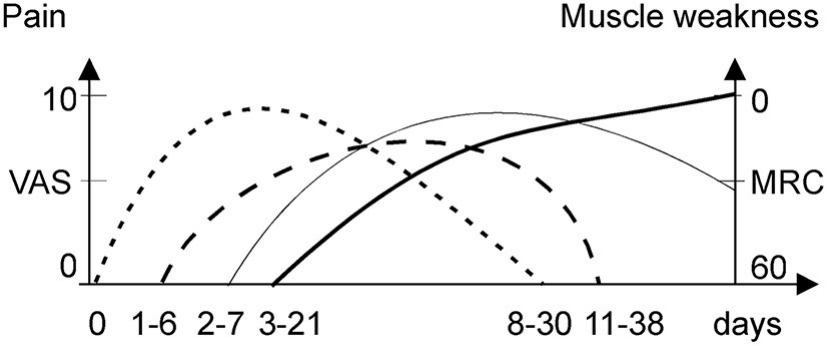
Onset and duration of pain syndromes and weakness during an acute attack. MRC, medical research council; VAS, visual acuity scale. Abdominal pain (dotted line), back pain (dash line), limb pain (thick solid line), and muscle weakness (thin solid line).

If an acute attack remains untreated or worsened by additional provoking factors (as noted above), it proceeds to acute neuropathy and/or encephalopathy, often termed a “severe acute attack” (14). If an acute attack is treated immediately, it is usually limited to abdominal pain, dysautonomia (tachycardia and elevated systolic blood pressure), and mild cognitive symptoms. The abdominal pain during acute attacks is severe either way, typically scored >9 on a visual analog scale (14). The neurological deficit usually develops after 3–21 days of abdominal pain and dysautonomia (Figure 1) (1). When progressive muscle weakness manifests, the intensity of abdominal pain may abate (7, 15), which may lead to a misdiagnosis of Guillain Barre syndrome (GBS). In recurrent attacks, with incomplete neurologic recovery, muscle weakness can start sooner after abdominal pain, or even precedes it (16).

#### Lower back pain

A third to a half of cases reported back pain mainly in the lumbar area (8, 17). The back and limb pain were pooled together in most studies with prevalence of 25% in mild sporadic attacks (18) and 50–70% in severe or mixed attacks suggesting partial association with neuropathy (5). Patients with recurrent attacks reported prevalence of limb over back pain during the acute attacks (12).

#### Limb pain

Limb pain is common (50–68%) and severe with acute porphyric neuropathy (5). It manifested as a severe deep myalgic or superficial neuropathic burning pain (12) lasting for 1–2 weeks (Figure 1). Porphyric neuropathy is predominantly motor (5, 7, 14) but may be associated with a proximal pattern sensory loss “old bath-costume distribution” differing from the classic length-dependent neuropathy (5, 7, 14). Paresthesia, dysesthesia, or hyperesthesia indicative of neuropathic pain are common (5, 7, 8, 13). Neuropathic pain is also common in acute attacks without overt motor weakness (6), especially in recurrent attacks (77%) (12). Several cases of rhabdomyolysis during acute attacks have been reported (19–21). However, the creatinine kinase levels in patient series were rarely reported, and thus, the frequency of rhabdomyolysis and its role in myalgia during attacks remains unclear.

#### Headache

Headaches are infrequent, yet can be severe, during an acute attack (7, 8, 22, 23). It is commonly associated with hypertension and has been reported with posterior reversible encephalopathy syndrome (PRES) (22–24). However, headaches are less common in porphyric PRES than in PRES of other origins, as AHP patients could be distracted by the exceedingly more severe abdominal pain (23).

### Between the attacks

Although most (82–94.4%) AHP patients do not feel pain outside the attacks (9, 17), some groups of patients frequently report mild to moderate chronic pain:

#### Patients with recurrent acute attacks

A prospective natural history study of 112 AHP patients experiencing recurrent attacks (≥3/year) or receiving prophylactic hemin treatment reported that chronic pain was present in 40% of patients (12). Similar percentages were reported in other series, and 25% of patients experienced daily pain (12, 25, 26). Chronic pain ranged from manageable with little impact, to having a significant impact on daily activities (26). Half of AHP patients were reported using opioids between attacks (12). In descending order, abdominal pain was the most frequent followed by headache, back pain, limb pain, and myalgia (12). The frequency of chronic pain did not differ significantly between patients who were on prophylactic hemin treatment and those who were not. Chronic pain mainly represented the neuropathic type based on burning and tingling sensations, poor response to pain medications, and partial loss of limb sensations, reported by 47% of patients with recurrent attacks during partial remission (26). In some cases, it was diffuse, involving the whole abdomen or the “entire body” (25, 26). The overlap of sensory (19%) and motor neuropathy signs (22%) (27) and chronic pain (25–40%) in these patients was common (12, 25, 26).

#### Delayed recovery from porphyric neuropathy

Recovery from acute porphyric neuropathy may take up to 2 years and is frequently complicated by chronic neuropathic pain associated with numbness, but detailed data are lacking (14).

## Diagnostic testing

### During acute attacks

#### Urine PBG and ALA testing

Any patient with recurrent severe acute abdominal pain with no obvious explanation should be screened for AHPs, especially if associated with neuropathic pain, hyponatremia, autonomic manifestations, or encephalopathy. An acute attack can be effectively diagnosed when the spot urine PBG, per mg urine creatinine, is markedly elevated. Note, that “urine porphyrins” may not be increased during the attacks and should not be first-line tests (15). DNA testing confirms the diagnosis and determines the AHP type.

#### Nerve conduction studies and electromyography

NCS-EMG studies typically show diffuse, motor-predominant, axonal neuropathy in patients with acute porphyric neuropathy. They help exclude other differential diagnoses (e.g., GBS, etc.) when associated with normal cerebrospinal fluid protein (28). It is still mandatory to exclude AHPs by testing urine PBG in any acute painful neuropathy (29).

### Between the attacks

AHP patients with chronic porphyric neuropathy symptoms may present to neurologists for evaluation of small fiber neuropathy (SFN). Diagnostic testing for AHPs should be considered for patients with a previous history of acute/subacute neuropathy, frequent emergency room visits with abdominal pain, and behavioral changes (15).

#### Laboratory testing

It is often challenging to distinguish flares of chronic pain from acute attacks based on clinical assessment without biochemical studies (2). Urinary PBG decreases between attacks, but may remain increased for years, without symptoms, in AIP patients (30), typically increasing ≈2-fold with a new attack. Urinary ALA is always increased during acute attacks and remains elevated in ~ 62% of AIP cases between attacks (31). In contrast, in patients with HCP or VP, the levels of urinary ALA and PBG are less significantly increased during an acute attack and fall quickly when the attack resolves (15).

#### NCS-EMG

Electrodiagnostic testing lends objective evidence to chronic large fiber neuropathy as a sequela of acute neuropathy in cases of chronic neuropathic pain (28). While the encephalopathy and acute autonomic symptoms resolve quickly after starting treatment, recovery of the neuropathy from an electrophysiologic standpoint is slower, taking months, often with incomplete normalization of motor and sensory amplitudes (32). Earlier electrodiagnostic studies did not correlate the severity of the electrodiagnostic findings with disease duration or number of previous attacks (28).

#### Histopathologic studies

The main nerve biopsy findings in porphyric neuropathy are non-specific including axonal loss and Wallerian degeneration. Grouped demyelination was observed in association with axonal degeneration and is likely secondary to the primary axonopathy. Pure sensory nerves are often intact (33). Given this lack of specificity and the motor predominance in porphyric neuropathy, sural nerve biopsy is not needed, unless the indication is to exclude another differential diagnosis (28).

Skin punch biopsy helps confirm a diagnosis of SFN in chronic neuropathic pain when NCS are normal (34). Hseih et al. performed serial skin biopsies on an AIP patient during and right after an attack. They reported reduced intraepidermal nerve fiber density (IENF), on day 48 from onset that did not improve as of day 92 despite improving muscle strength. These findings underscore a potential role of SFN in the chronic pain patients develop between attacks (35).

#### Autonomic studies

Autonomic testing could provide evidence of functional impairment of the small nerve fibers. Evidence of cardio-vagal impairment between attacks has been reported in a cohort of genetically unconfirmed AIP patients (36). The assessed cardio-vagal parameters included the heart rate responses to deep breathing (HRDB), and Valsalva maneuver. The Valsalva ratio was normal during remission, whereas HRDB remained reduced (36). This discrepancy may reflect the higher sensitivity of HRDB (28). Another study used frequency-domain spectral analysis of heart rate variability and reported similar findings in a genetically confirmed Swedish AIP cohort (37).

## Pathophysiology of pain

The peripheral nociceptors are axon terminals from small dorsal root ganglion (DRG) neurons that innervate somatic, vascular, and visceral structures. These neurons project to the dorsal horn and use L-glutamate as their primary neurotransmitter (38). ALA has structural similarities to GABA and glutamate and, thus, may serve as their receptor agonist or antagonist in a dose-dependent manner (39). Excess ALA causes mitochondrial impairment and decreased neuronal ATP production leading to dysfunctional fast axonal transport (40) in addition to mitochondrial and nuclear DNA damage in other cell lines (41). Elevated ALA levels are pro-oxidative *in-vitro* and are associated with increased formation of reactive oxygen species in neural tissue which could damage Schwann cells (42, 43).

The different subtypes of nociceptive DRG neurons express different ion channels and receptors; have different thresholds of activation by painful mechanical, thermal, or chemical stimuli; and terminate in different laminae of the dorsal horn, reflecting the complexity of pain pathways (44). Neuropathic pain is often a consequence of a primary lesion affecting these peripheral nociceptive axons or central pain pathways at the levels of the spinal cord, brainstem, or thalamus (45). This is different from inflammatory nociceptive pain that occurs in response to tissue injury. The nociceptive and the autonomic neural circuits share important features: (1) they are mediated by thin-myelinated and/or unmyelinated nerve fibers; (2) they respond to injury and show functional plasticity in response to local inflammation; (3) they involve overlapping areas and pathways in the central nervous system; and (4) their activity is influenced by emotional factors (46).

### During acute attacks

#### Abdominal pain

The exact mechanisms of abdominal pain are still unclear (28). Autonomic neuropathy is likely responsible for most symptoms during attacks including the abdominal pain (5, 36). This was supported by finding occasional intestinal spasms alternating with dilatations without structural abnormalities in diagnostic laparotomies (7, 8, 13) and autopsy reports of vagus nerve demyelination, axonal loss, and chromatolysis of sympathetic ganglia (47). The blood-nerve-barrier (BNB) is less restrictive in the autonomic ganglia and absent at free nerve endings, including gastrointestinal small nerves (5, 36, 48), rendering these sites more vulnerable to ALA neurotoxic effects (28). ALA also has a direct gut-spasmodic effect (48) which is likely mediated by the gastrointestinal 5-HT receptors (49). Disturbance of tryptophan metabolism during acute attacks (50, 51) could contribute to the abnormal gut motility *via* 5-HT_1A_, 5-HT_3_, 5-HT_4_, and 5-HT_7_ receptors (52). Other mechanisms like intestinal vasoconstriction and ischemia were also suggested (53, 54).

#### Non-abdominal pain

Large (7, 32, 55) and small fiber neuropathy (35) were confirmed in cases with acute motor-predominant porphyric neuropathy. The prevalence of back and limb pain (70%) over muscle weakness (50%) in a some cohorts of acute attacks (56) suggested additional mechanisms of non-abdominal pain, other than limb neuropathy. The finding of low back pain preceding limb pain (Figure 1) suggests that the nerve roots are a primary site of pathology, evidenced by relative sparing of sensory NCS. Thus, porphyric neuropathy, similar to GBS, behaves like a polyradiculoneuropathy. Nerve roots, like autonomic ganglia, have less restrictive BNB and are more vulnerable to the toxic effect of ALA (28).

### Between the attacks

**Figure 2:**
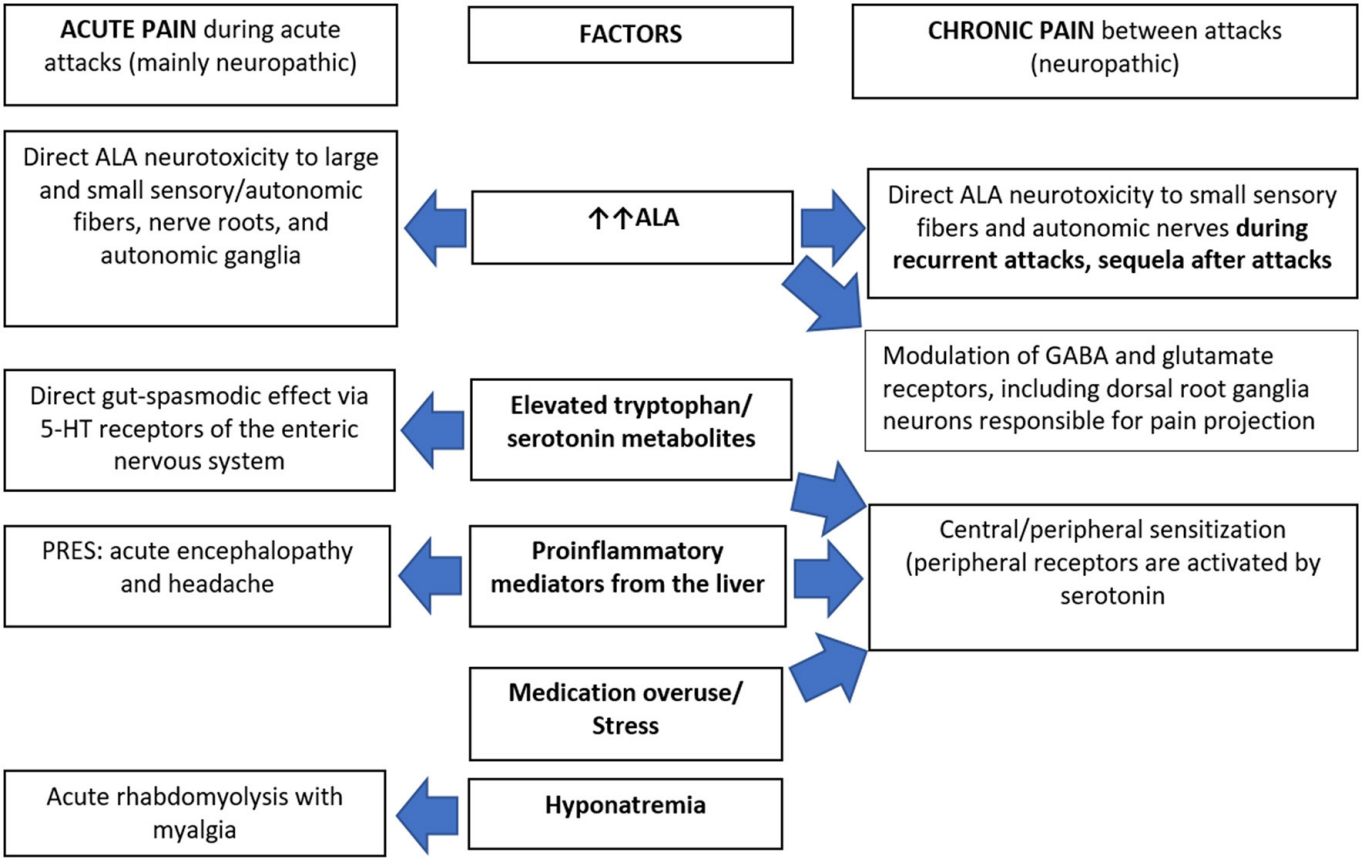
Pathophysiology of pain in acute hepatic porphyrias. 5-HT, 5-hydroxy tryptamine (serotonin); ALA, δ-aminolaevulinic acid; GABA, gamma aminobutyric acid; PRES, posterior reversible encephalopathy syndrome.

The potential explanations of chronic pain in AHPs (Figure 2) could include on-going chronic SFN due to ALA neurotoxicity in “high-ALA excretors.” Givosiran therapy dramatically decreased ALA levels and reduced pain severity in AIP patients, but many still experienced chronic pain suggesting slow recovery of the neuropathy in patients with frequent attacks (57). Chronic peripheral sensitization reflects functional up-regulation of some sodium channels including Na v1.7, Na v1.8, and Na v1.9 on the free nerve endings. This could be elicited by proinflammatory mediators from the liver that are significantly elevated in AIP patients with recurrent attacks (58, 59). Some mediators potentially induce glutamate-mediated activation of N-methyl D-aspartate receptors. This results in calcium influx triggering several downstream cascades that maintain a state of hyperexcitability of the projecting neuron. This process is known as central sensitization (60) and is further enhanced by pain medication overuse.

## Management of pain

### During acute attacks

Specific treatment of the first acute attacks with heme preparations improves severe pain, dysautonomia, and neuropathy progression within a few days (1, 18, 61). Recurrent attacks can be prophylactically decreased or prevented with heme infusions or monthly subcutaneous givosiran (see below). Since abdominal pain is often intense (14), opiates are needed immediately. Doses are individualized, based on risk of adverse outcomes, prior effective doses, co-morbidities, concomitant medications, and response to therapy. Immediate-release opioids, including morphine, meperidine, oxycodone, tramadol, and fentanyl have been used without complication (1). Benzodiazepines, such as lorazepam, can be used in inpatient settings to potentiate the analgesic effect and to decrease the concomitant anxiety (61). Gabapentin and pregabalin should be used in acute attacks where clear neuropathic features are present. Stretching, sensory reintegration, and psychotherapy should be included in any rehabilitation program for porphyric neuropathy to prevent chronic pain. Opiates should be replaced with less addictive analgesics as early as possible and should be avoided for long-term use after discharge (61).

### Between the attacks

#### Symptomatic pain management

Pharmacologic treatments of chronic neuropathic pain, in general, include certain antidepressants and antiepileptics (62). Antidepressants, like serotonin–noradrenaline reuptake inhibitors (duloxetine 60–120 mg/d, venlafaxine 150–225 mg/d) and tricyclic agents e.g., amitriptyline 25–150 mg/d are recommended first-line (63). Antiepileptics like pregabalin (150–600 mg/d) and gabapentin (1,200–3,600 mg/d) are also generally recommended first-line treatments by most scientific societies (63, 64). There is a potential risk of abuse with these antiepileptics and increased respiratory depression when combined with high-dose opioids (62). All the above-mentioned medications are deemed safe in porphyria ^1^. Table 1 includes porphyria-specific safety profiles of commonly used medications (28).

**Table 1:** Safety profiles of commonly prescribed neurologic medications (28).

	**Safe/probably safe**	**Probably unsafe**	**Unsafe**
**Anti-epileptic drugs**			
Carbamazepine, Phenobarbital, Valproic acid, Phenytoin, Felbamate			+
Oxcarbazepine, Eslicarbazepine, Ethosuximide, Topiramate, Perampanel		+	
Lamotrigine, Lacosamide, Zonisamide, Vigabatrin, Levetiracetam, Clonazepam	+		
**Neuropathic pain drugs**			
Gabapentin, Pregabalin, Amitriptyline	+		
Duloxetine, Venlafaxine	+		
Opioids (except for Dextropropoxyphene)	+		
**Benzodiazepines**			
Diazepam, Lorazepam, Oxazepam, Midazolam	+		
Zopiclone, Zolpidem, Zaleplon	+		
**Antidepressants**			
Mirtazapine, Escitalopram, Citalopram, Fluvoxamine, Trazodone, Agomelatine	+		
**Neuroleptics**			
Chlorpromazine, Levomepromazine, Fluphenazine, Prochlorperazine	+		
Olanzapine, Clozapine, Trifluoperazine, Haloperidol, Droperidol, Sulpiride	+		
Thioridazine, Melperone, Risperidone		+	
**Analgesics**			
Ibuprofen, Ketoprofen, Naproxen, Celecoxib, Etoricoxib, Acetylsalicylic acid, Indomethacin	+		
Paracetamol	+		
Metamizole, Phenazone		+	
**Muscle relaxants**			
Baclofen	+		
Carisoprodol			+
Cyclobenzaprine		+	
**Antiparkinson drug**			
Levodopa, Pramipexole, Rasagiline, Ropinirole	+		
Others*			
Hydroxyzine	+		
Triptans	+		
Botulinum toxin	+		
Melatonin	+		

*Information on more drugs is available on http://porphyriadrugs.com/, and www.drugs-porphyria.org/index.php.

Chronic use of opioids was qualitatively studied in patients with recurrent attacks where all participants (*n* = 16) were prescribed narcotics. Most patients (56%) disliked taking them regularly due to side effects or concerns of abuse. Three participants reported struggling with addiction and felt they were not appropriately counseled (25). In general, tramadol (100–400 mg/d) and stronger opioids, like oxycodone and hydrocodone are moderately effective in peripheral neuropathic pain. These are recommended second and third-line, respectively, with careful evaluation of addiction risks (62–64). Naloxone should be prescribed for patients taking ≥ 50 mg morphine milligram equivalent a day (65). In general, we recommend treating mild to moderate chronic neuropathic pain (VAS<7) with antidepressants and gabapentoids. More severe pain can be treated with a short-term opioid regimen and/or heme on-demand infusions. NSAIDs and acetaminophen can be helpful with mild musculoskeletal pain, yet they are not recommended for neuropathic pain based on inefficacy and lack of evidence (63).

#### Healthy lifestyle modifications

These include adequate nutrition with achieving upper normal body mass index, cessation of smoking and alcohol, and stress-coping strategies. They should be implemented soon after diagnosis (1).

#### Prophylactic hemin infusions

Weekly, biweekly, or monthly IV hemin infusions have been used to reduce the frequency of attacks (9). A British audit of 22 patients with severe recurrent attacks who received 1–8 heme arginate infusions/month reduced pain frequency in 67% of patients. However, 12 patients continued to have repeated hospital admissions because of disease worsening, tachyphylaxis, or development of chronic pain (66).

#### siRNA silencing of ALAS1

Givosiran, an siRNA molecule directed against hepatic *ALAS1* mRNA, was approved by the FDA and the European Medicines Agency, following a 6-month randomized, double-blinded, placebo-controlled phase-3 study in AHP patients. Monthly subcutaneous injections of 2.5 mg/kg led to 74 and 77% reduction in the mean annual attack rate and decreased annualized days of IV hemin use, respectively. The daily worst pain score was significantly lower in the AIP givosiran group compared with placebo (67). Real-world experience showed that givosiran prevented recurrent attacks in many patients and is most effective when given early in the disease course (57). Long-term experience will determine the effect of givosiran on improving the chronic pain in AHP patients.

#### Liver transplantation

Liver transplantation is curative for patients with severe recurrent attacks where medical management failed or caused significant complications (68, 69). It led to cessation of attack recurrence and improvement in chronic pain (70). However, the introduction of safer prophylactic therapies has greatly reduced the need for liver transplantation (28).

## Conclusion

While acute abdominal pain, likely mediated by autonomic neuropathy, is a key symptom of AHP acute attacks, chronic pain is common and contributes to the disease burden. Chronic pain is mainly neuropathic and potential mechanisms include ongoing SFN, delayed recovery of acute neuropathy, and central and/or peripheral sensitization. ALA plays a major role in both acute and chronic pain *via* its neurotoxic effect, especially where the BNB is less restrictive or absent, i.e., the autonomic ganglia, nerve roots, and free nerve endings. Chronic high ALA excretors likely have chronic ALA-induced neuropathy. Severe episodic acute pain should be treated with opiates combined with hemin infusions, whereas recurrent acute attacks should be treated with monthly givosiran injections. Symptomatic treatment of chronic pain should start with gabapentinoids and certain antidepressants before opiates. Givosiran reduces ALA and PBG levels and likely has long-term benefits for chronic pain. Skin punch biopsy for IENF and autonomic studies could demonstrate if small fiber/autonomic neuropathy is common in chronic AHPs. Animal models for the disease could provide more information on whether potential pain mechanisms recently described in other neuropathies are also important in AHPs.

## Author contributions

MK: writing and editing the original draft. EP: writing and editing the original draft and figures. RD: reviewing and editing the final manuscript version. All authors contributed to the article and approved the submitted version.

## Funding

The open access publication fee for this manuscript was funded by the NIH Porphyria and Rare Disease Consortium (U54DK083909).

## Conflict of interest

Author MK received consulting fees from Alnylam Pharmaceuticals. Author EP received consulting fees from Alnylam Pharmaceuticals and Recordati Rare Diseases. Author RD is a consultant for Alnylam Pharmaceuticals and Recordati Rare Diseases. He has received grants from both entities. He receives royalties for a licensed patent to Alnylam Pharmaceuticals.

## Publisher's note

All claims expressed in this article are solely those of the authors and do not necessarily represent those of their affiliated organizations, or those of the publisher, the editors and the reviewers. Any product that may be evaluated in this article, or claim that may be made by its manufacturer, is not guaranteed or endorsed by the publisher.

## Footnotes

^1^ http://www.drugs-porphyria.org/
